# Association between the triglyceride glucose index and obstructive sleep apnea and its symptoms: results from the NHANES

**DOI:** 10.1186/s12944-024-02125-w

**Published:** 2024-05-06

**Authors:** Chao Wang, Mengdi Shi, Chunsheng Lin, Jingyi Wang, Liangzhen Xie, Yan Li

**Affiliations:** 1 Heilongjiang University of Traditional Chinese Medicine, Harbin, 150006 China; 2 The Second Affiliated Hospital of Heilongjiang University of Traditional Chinese Medicine, Harbin, 150001 China; 3 The First Affiliated Hospital of Heilongjiang University of Traditional Chinese Medicine, Harbin, 150040 China

**Keywords:** Obstructive sleep apnea, Insulin resistance, NHANES, Receiver operating characteristic, Triglyceride glucose index

## Abstract

**Background:**

Certain studies have indicated a link between obstructive sleep apnea and insulin resistance in specific populations. To gain more clarity, extensive research involving a broad sample of the overall population is essential. The primary objective of this study was to investigate this correlation by utilizing data from the National Health and Nutrition Examination Survey database.

**Methods:**

The analysis incorporated data from the National Health and Nutrition Examination Survey database spanning the time periods from 2005 to 2008 and from 2015 to 2018, with a focus on American adults aged 18 years and older after applying weight adjustments. Key variables such as obstructive sleep apnea, triglyceride glucose index, and various confounding factors were considered. A generalized linear logistic regression model was used to investigate the association between obstructive sleep apnea and the triglyceride glucose index, with additional exploration of the consistency of the results through hierarchical analysis and other techniques.

**Results:**

The study included participants aged between 18 and 90 years, with an average age of 46.75 years. Among the total sample, 50.76% were male. The triglyceride glucose index demonstrated a diagnostic capability for obstructive sleep apnea, with an AUC of 0.701 (95% CI: 0.6619–0.688). According to the fully adjusted model, individuals in the fourth quartile of the triglyceride glucose index showed an increased likelihood of having obstructive sleep apnea compared to those in the first quartile (OR: 1.45; 95% CI: 1.02–2.06; *P* < 0.05). Subgroup analysis indicated that male sex (OR: 2.09; 95% CI: 1.76–2.45; *P* < 0.05), younger age (OR: 2.83; 95% CI: 2.02–3.96; *P* < 0.05), white ethnicity (OR: 2.29; 95% CI: 1.93–2.73; *P* < 0.05), and obesity (OR: 1.54; 95% CI: 1.28–1.85; *P* < 0.05) were correlated with an elevated risk of OSA.

**Conclusions:**

This study demonstrated a strong association between an elevated TG index and OSA. Additionally, the triglyceride glucose index could serve as an independent predictor of obstructive sleep apnea.

## Background

Sleep-related disorders have a substantial impact on an individual’s daily activities and overall quality of life. Nevertheless, among American adults aged 20–79 years, 16.3% reported of experiencing sleep problems [1]. Undoubtedly, sleep apnea syndrome is considered one of the most severe sleep disorders. In the United States, the prevalence of obstructive sleep apnea (OSA) among adults is as high as 25% [2]. Clinical symptoms of OSA syndrome include nighttime snoring accompanied by apnea episodes and daytime drowsiness. The primary consequence of this disorder is a heightened susceptibility to motor vehicle and workplace accidents [3]. OSA syndrome is characterized by repetitive collapses of the pharynx during sleep, thus resulting in inadequate oxygen levels as oxygen saturation decreases by more than 3% from baseline. Although these events are brief (lasting 3–15 s), patients often remain asleep; however, frequent oxygen desaturation leads to disrupted sleep patterns. Intermittent hypoxia plays a crucial role in the pathophysiology and outcomes of apnea and hypoventilation, thus contributing to symptoms such as excessive daytime sleepiness (EDS), cardiovascular complications (CVD), and an elevated risk of mortality from any cause [4]. Furthermore, reduced oxygen levels can readily trigger the sympathetic nervous system, thus resulting in heightened oxidative stress and inflammation, thereby significantly elevating the risk of CVD [5, 6]. Multiple studies have demonstrated compelling evidence of a substantial correlation between OSA and an increased incidence of high-risk CVD [7–9]. Failure to promptly treat patients with OSA significantly increases the risk of developing cardiovascular and cerebrovascular complications. Given the intricate diagnostic process for OSA, conclusive diagnosis necessitates instrumental and other diagnostic assessments. Additionally, the prevention and management of this condition have consistently posed challenges, which are largely due to an insufficient focus on OSA [10].

Consequently, the identification of the risk factors for OSA is urgently needed in clinical practice.

Insulin resistance triggers heightened insulin secretion to regulate glucose levels, thus leading to persistent hyperinsulinemia, which subsequently causes increased oxidative stress and inflammatory reactions [11]. Insulin resistance is a characteristic feature of diabetes mellitus (DM), hypertension, and specific cardiovascular conditions, including stroke [12, 13]. Hence, the swift and precise assessment of insulin resistance is vital in clinical settings. Studies have demonstrated a significant association between the triglyceride-glucose index and the occurrence of insulin resistance, thus establishing it as a straightforward and dependable surrogate marker for insulin resistance [14–16]. Alongside the Homeostasis Model Assessment of Insulin Resistance, the triglyceride-glucose (TyG) index is another gold standard for assessing insulin resistance. However, the TyG index is notable because it does not necessitate insulin measurements, thus reducing the cost of the technique and enhancing its clinical feasibility. Furthermore, the TyG index has a wide range of applications [17].

Recent research has suggested a link between OSA and conditions such as diabetes and hypertension [18, 19]. Moreover, there is a close relationship between the TyG index and these diseases [20–22]. A recent meta-analysis indicated that the TyG index serves as a readily measurable indicator of insulin resistance and is employed in the assessment of OSA for both diagnostic and prognostic purposes. Patients with OSA exhibit significantly greater TyG index levels than healthy controls [23]. The TyG index may be an independent risk factor for OSA [24].

This study investigated whether the TyG index is an independent influencing factor of OSA. In addition, this study aimed to determine whether the TyG index can serve as a predictive factor for OSA. To evaluate the correlation between the TyG index and OSA incidence, data from the National Health and Nutrition Examination Survey (NHANES) were collected.

## Methods

### Study participants

**Fig. 1:**
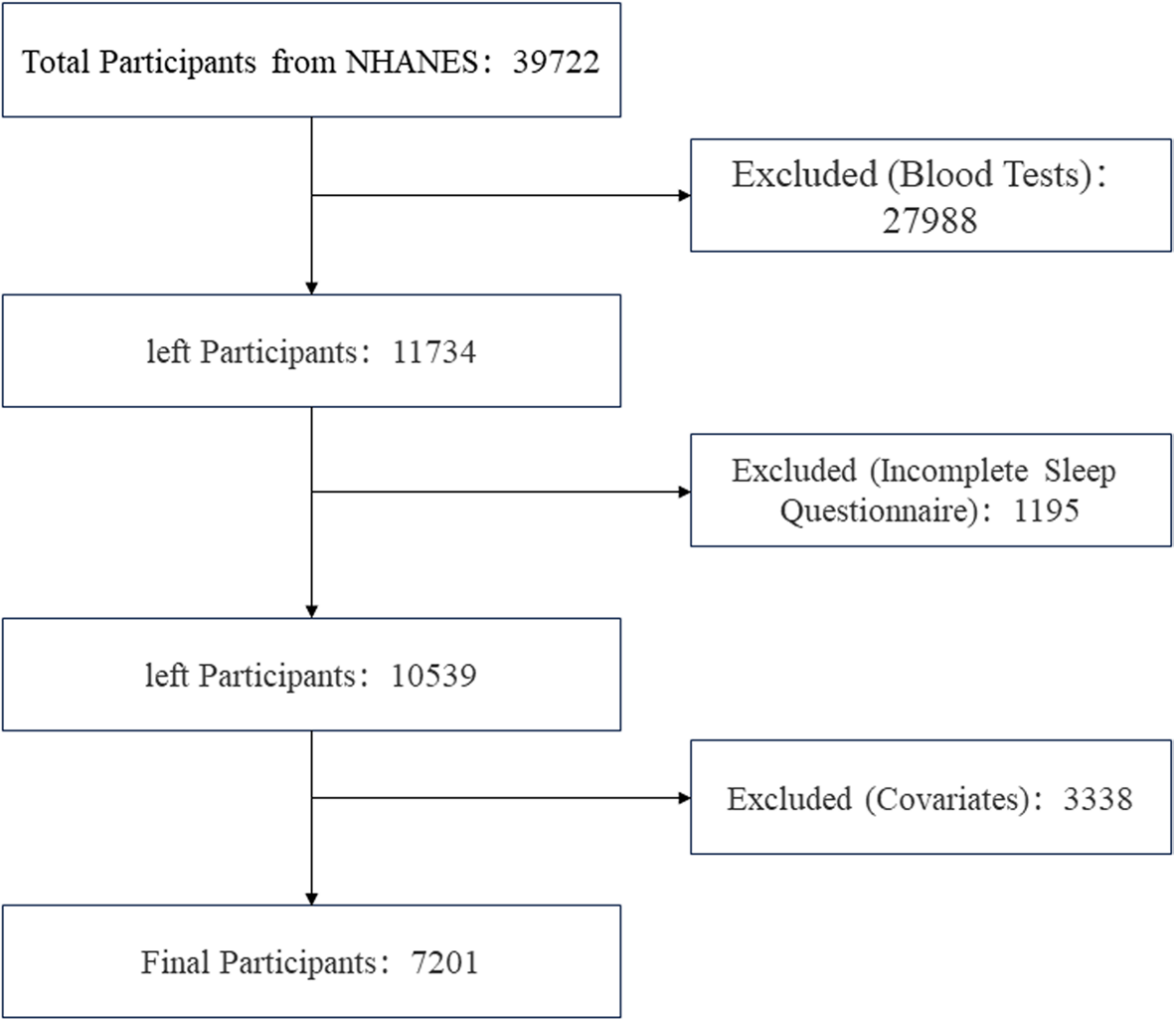
Selection of the study population

The objective of this study was to explore the relationship between OSA and the TyG index. Participants were sourced from the NHANES datasets spanning from 2005–2008 and from 2015–2018, as the relevant sleep questionnaires were limited to these time periods. From a total of 39,722 participants, 27,988 individuals lacking fasting glucose and triglyceride data were excluded, along with 1,195 participants who did not fully complete the sleep questionnaires. Additionally, 3,338 participants with incomplete demographic and health-related data, such as age, sex, race, body mass index, poverty ratio, hypertension status, diabetes mellitus status, and cardiovascular disease status, were excluded from the analysis. Ultimately, the study included 7,201 participants, as illustrated in Fig. 1.

### Data collection

Health-related interviews were meticulously conducted at the participants’ residences, whereas blood samples were collected at conveniently located mobile examination units. The collected information encompassed a wide array of demographic and lifestyle factors, including age categorized into four groups (18–34, 35–48, 49–63, and 64–85 years); gender classified as male or female; race or ethnicity identified as white, black, or other; smoking status categorized as never smoked, former smoker, or current smoker; and alcohol consumption indicated as a yes or no response. Additionally, body mass index (BMI, calculated in kg/m2) was determined from the provided physical measurements. This BMI calculation allowed for the classification of participants into three weight categories: normal for a BMI below 25, overweight for a BMI between 25 and 30, and obese for a BMI of 30 or higher [4].

### The OSA status, triglyceride glucose index, and other covariates were evaluated

#### Obstructive sleep apnea syndrome

The assessment of OSA included adjustments for the multivariate apnea prediction index (MAPI) and variables obtained from the NHANES. To mimic the original MAPI for predicting sleep issues, two questions related to the frequency of "snoring" and "snoring stopping breathing" were modified in the NHANES dataset. Similar to the original MAPI, the apnea index was computed as the average of the nonmissing responses, with factors such as BMI, age, and sex incorporated into the revised multivariate apnea prediction model. The adapted multivariate apnea prediction model exhibited strong test–retest reliability after a two-week interval (correlation coefficient = 0.92). A threshold of 50% or greater on the adapted multivariate apnea prediction yielded a sensitivity of 88% (95% CI: 84%-92%) but a lower specificity of 55% (95% CI: 48%-62%) [25, 26].

#### Triglyceride glucose index

The TyG index was computed by using the following formula: Ln (fasting triglycerides [mg/dl] × fasting blood glucose [mg/dl]/2) [27, 28].

#### Other covariables

The selection of covariates was guided by previous research and theoretical rationale. Diabetes mellitus was characterized by a diabetes diagnosis, the use of diabetes medication, or the use of insulin. CVD encompasses conditions such as coronary heart disease, congestive heart failure, heart attack, stroke, and angina, which were categorized as being either present or absent. Hypertension was ascertained through a combination of questionnaire responses and blood pressure readings. The questionnaire inquired about a health care professional’s diagnosis of high blood pressure and current medication usage. Hypertension was defined as a systolic blood pressure exceeding 140 mmHg or a diastolic blood pressure surpassing 90 mmHg. Three individuals were diagnosed with hypertension based on these criteria. Blood pressure measurements (both systolic and diastolic) were conducted at the mobile examination center [29–31].

### Statistical analyses

In the analysis of the NHANES data, this study followed the guidelines outlined by the NHANES. This study incorporated interview weight variables (WTINT2YRs) in all of This analyses to guarantee nationally representative estimates. To account for standard errors (SEs) associated with complex survey designs, this study utilized the primary sampling unit variable (SDMVPSU) and the stratification variable (SDMVSTRA).

Continuous variables are summarized as the means with 95% confidence intervals (95% CIs) or medians with interquartile ranges, depending on the variable distribution, whereas categorical variables are depicted as counts and proportions. Subsequent analyses involved multivariate logistic regression and linear models to evaluate the impact of the TyG index on OSA and its symptoms. The crude model remained unadjusted, whereas Model 1 included adjustments for age, sex, race, PIR, and BMI. Model 2 further accounted for variables in Model 1, including hypertension, CVD incidence, smoking status, alcohol consumption, and DM. The area under the receiver operating characteristic (ROC) curve was calculated without covariate adjustments. Subgroup analyses were stratified and presented by using a fully adjusted Model 3. Nonlinear relationships between TyG index levels and obstructive sleep apnea symptoms were evaluated by using restricted cubic spline curves.

Thorough statistical analyses were conducted by using the R software suite (version 4.1.2). Statistical significance was determined by *P* values less than 0.05.

## Results

### Baseline characteristics of the participants

Table 1 presents the distribution of clinical characteristics among the study participants, with an average age of 46.75 years and males comprising 50.76% of the sample. Non-Hispanic Whites were the predominant ethnic group at 68.41%. Significant differences were observed across all of the parameters except for daytime sleepiness.

**Table 1:** Weighted baseline characteristics of the participants

Characteristics	All Participants n (%)	High-risk n (%)	Low-risk n (%)	*P-*value
Age	46.75(0.40)	54.18(0.41)	42.49(0.45)	< 0.0001
FBG	107.27(0.47)	116.50(0.88)	101.98(0.42)	< 0.0001
TG	128.04(1.67)	155.44(2.95)	112.32(1.69)	< 0.0001
TyG	8.63(0.01)	8.91(0.02)	8.47(0.01)	< 0.0001
TyG quartile				< 0.0001
Q1	1804(25.74)	348(11.73)	1456(33.78)	
Q2	1797(25.23)	620(21.32)	1177(27.47)	
Q3	1799(24.99)	802(29.12)	997(22.63)	
Q4	1801(24.03)	1006(37.83)	795(16.11)	
Sex				< 0.0001
Female	3487(49.24)	662(24.10)	2825(63.65)	
Male	3714(50.76)	2114(75.90)	1600(36.35)	
Race				< 0.0001
Mexican American	1217(8.40)	443(7.70)	774(8.80)	
Non-Hispanic Black	1491(10.32)	578(9.69)	913(10.68)	
Non-Hispanic White	3032(68.41)	1266(71.68)	1766(66.53)	
Other Hispanic	707(5.28)	291(5.23)	416(5.30)	
Other Race	754(7.60)	198(5.70)	556(8.68)	
PIR				0.01
< = 1	1231(11.03)	433(10.19)	798(12.72)	
> 1	5364(82.52)	2127(89.81)	3237(87.28)	
BMI				< 0.0001
Normal	2126(30.82)	246(7.38)	1880(44.26)	
Obese	2666(36.28)	1720(64.87)	946(19.88)	
Overweight	2409(32.91)	810(27.76)	1599(35.86)	
Trouble sleeping				< 0.0001
No	5377(72.49)	1944(67.12)	3433(75.57)	
Yes	1824(27.51)	832(32.88)	992(24.43)	
Sleep duration				< 0.001
< 7	2233(28.15)	952(31.53)	1281(26.22)	
> 9	509(5.89)	171(5.04)	338(6.38)	
7–9	4459(65.96)	1653(63.43)	2806(67.40)	
Snore				< 0.0001
No	1966(26.30)	230(6.49)	1736(37.66)	
Yes	5235(73.70)	2546(93.51)	2689(62.34)	
Stop breathing				< 0.0001
No	5466(76.00)	1546(54.33)	3920(88.42)	
Yes	1735(24.00)	1230(45.67)	505(11.58)	
Daylight Sleepy				0.3
No	1873(20.94)	706(20.17)	1167(21.38)	
Yes	5328(79.06)	2070(79.83)	3258(78.62)	
Hypertension				< 0.0001
No	4278(63.89)	1166(46.04)	3112(74.12)	
Yes	2923(36.11)	1610(53.96)	1313(25.88)	
CVD				< 0.0001
No	6262(90.27)	2287(86.19)	3975(95.16)	
Yes	756(8.02)	479(13.81)	277(4.84)	
DM				< 0.0001
No	4888(72.48)	1399(54.57)	3489(82.75)	
Yes	2313(27.52)	1377(45.43)	936(17.25)	
Smoke				< 0.0001
Former	1761(25.83)	944(33.65)	817(21.35)	
Never	3962(54.11)	1294(48.16)	2668(57.52)	
Now	1478(20.06)	538(18.19)	940(21.13)	
Alcohol.user				< 0.001
No	1029(10.86)	280(7.87)	749(12.58)	
Yes	6172(89.14)	2496(92.13)	3676(87.42)	

*Abbreviations*: *FBG* Fasting blood glucose, *TG* Triglyceride, *TyG* Triglyceride-glucose index, *CVD* Cardiovascular disease, *DM* Diabetes mellitus

Continuous variables are shown as the mean (SD), and categorical variables are shown as percentages

### Receiver operating characteristic of the TyG index to OSA and OSA symptoms

**Fig. 2:**
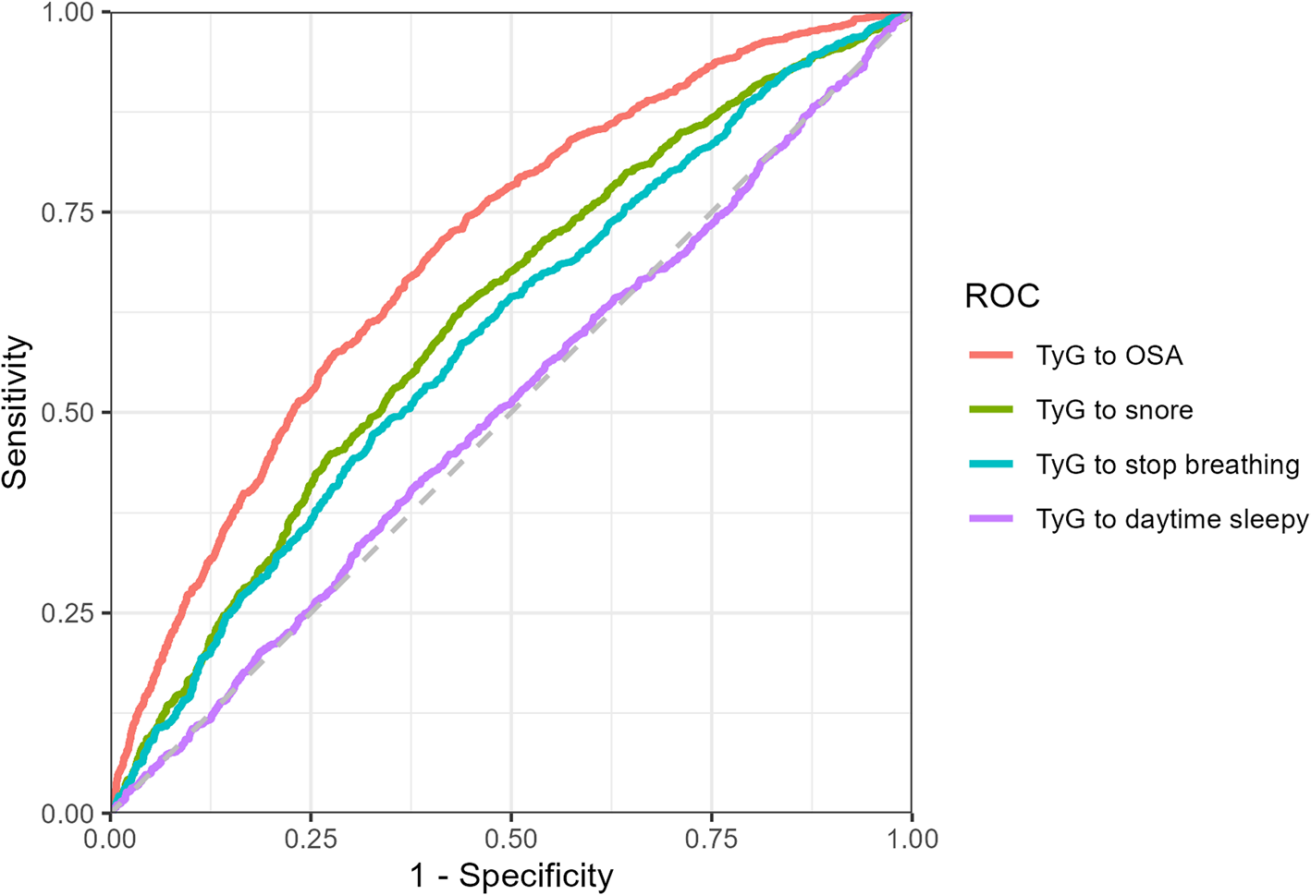
ROC curve for OSA and OSA symptoms

Figure 2 shows the population-weighted ROC curve for OSA and its symptoms. The area under the curve (AUC) values for the TyG index were as follows: 0.618 (95% CI: 0.5851–0.6161) for snoring, 0.594 (95% CI: 0.558–0.5897) for cessation of breathing, and 0.505 (95% CI: 0.4822–0.5138) for daytime sleepiness. Notably, TyG exhibited effective discrimination of OSA, with an AUC of 0.701 (95% CI: 0.6619–0.688).

### Associations between the TyG index and OSA and OSA symptoms

#### Associations between the TyG index and OSA

**Fig. 3:**
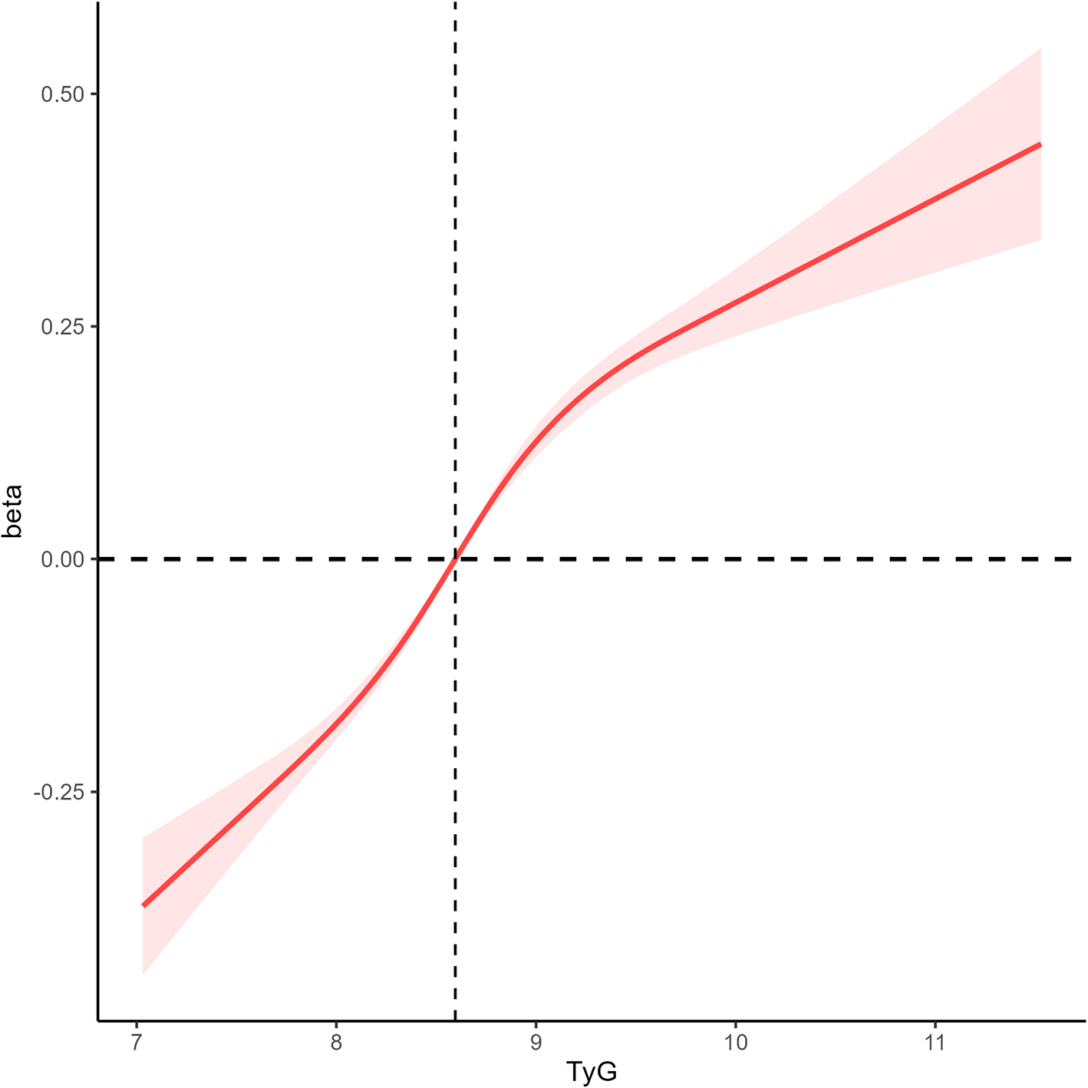
Restricted cubic spline fitting for the association between TyG index levels and OSA

As shown in Fig. 3, there was a linear relationship between the TyG index and OSA.

In Table 2, within the final model, a higher TyG index was associated with an increased relative risk of OSA (OR = 0.02; 95% CI: 0.00–0.04). Compared to patients in the first quartile of the TyG index, patients in the fourth quartile had a greater multivariate-adjusted OR for having OSA (OR = 1.45; 95% CI: 1.02–2.42).

**Table 2:** Weighted relationship between the triglyceride-glucose index and OSA

**Exposure**	Crude model		Model 1		Model 2	
	95% CI	*P -*value	95% CI	*P -*value	95% CI	*P -*value
TyG index	0.24(0.22,0.26)	< 0.0001	0.04(0.02,0.06)	< 0.001	0.02(0.00,0.04)	0.02
TyG index quartile						
Q1	1		1		1	
Q2	2.20(1.66,2.91)	< 0.0001	1.02(0.68, 1.53)	0.92	1.00(0.67, 1.50)	0.99
Q3	3.79(3.06,4.70)	< 0.0001	1.17(0.84, 1.64)	0.35	1.09(0.77, 1.54)	0.61
Q4	7.10(5.55,9.06)	< 0.0001	1.69(1.18, 2.42)	0.01	1.45(1.02, 2.06)	0.04
*P* for trend		< 0.0001		0.001		0.02

The data are presented as odds ratios (ORs), 95% confidence intervals (CIs), and *P* values

Crude model: unadjusted

Model 1: adjusted for sex, race, age, BMI, and PIR

Model 2: adjusted for Model 1 and hypertension, CVD, smoking status, alcohol consumption, and DM

In Table 3, within the crude model, the TyG index was related to Stop breathing and Snoring. In Model 1, a higher TyG index was associated with an increased relative risk of snoring. Moreover, in this study, daylight sleepiness and the TyG index were not significantly related.

**Table 3:** Relationships between the triglyceride-glucose indices and OSA symptoms

**Exposure**	Crude model		Model 1		Model 2	
	95% CI	*P -*value	95% CI	*P -*value	95% CI	*P -*value
Daylight sleepy	0(-0.01,0.02)	0.59	0.01(-0.01, 0.03)	0.24	0.01(-0.02, 0.03)	0.61
Stop breathing	0.09(0.07,0.11)	< 0.0001	0.04(0.02,0.06)	< 0.001	0.03(0.01,0.06)	0.01
Snore	0.11(0.09,0.13)	< 0.0001	0.03(0.00,0.06)	0.02	0.02(0.00, 0.05)	0.08

The data are presented as odds ratios (ORs), 95% confidence intervals (CIs), and *P* values

Crude model: unadjusted

Model 1: adjusted for sex, race, age, BMI, and PIR

Model 2: adjusted for Model 1 and hypertension, CVD, smoking status, alcohol consumption, and DM

#### Associations between the TyG index and OSA

**Fig. 4:**
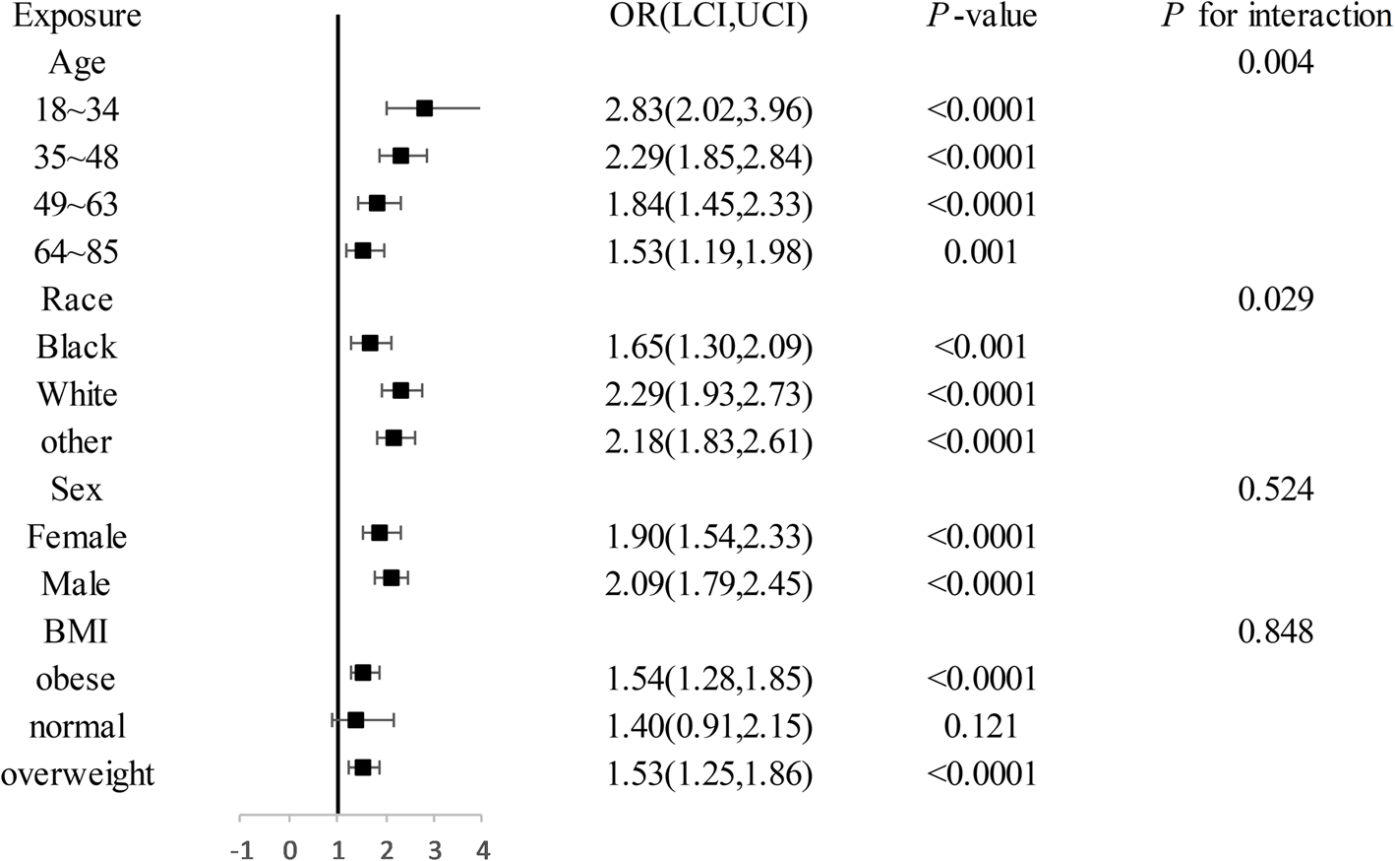
Odds of OSA subgroups based on the increasing TyG index with various demographic variables

Subgroup analysis was performed to assess the strength of the relationship between the TyG index and OSA. Stratified analysis findings indicated that younger age, white ethnicity, male sex, and obese individuals exhibit heightened sensitivity to the TyG index (refer to Fig. 4 for specific data). Conversely, it appeared that sensitivity to the TyG index decreased with advancing age. Adjusted for hypertension, CVD, smoking status, alcohol use status, and DM status. Figure 4 adjusted for hypertension, CVD, smoking status, alcohol use status, and DM status.

Moreover, compared to individuals in the first quartile of the TyG index, younger participants, individuals of other races, females, and obese individuals in the fourth quartile appeared to exhibit greater vulnerability to OSA (refer to Table 4 for specific data). This observation aligns with the correlation between the TyG index and OSA. Furthermore, Table 4 illustrates that an elevated TyG index was linked to a greater probability of OSA.

**Table 4:** OSA subgroups based on the TyG index quartile with various demographic variables

Character	Q1	Q2	Q3	Q4	*P* for trend	*p* for interaction
Age						0.008
18 ~ 34	ref	2.247(1.185, 4.262)	3.525(1.866, 6.660)	6.421(3.325,12.400)	< 0.0001	
35 ~ 48	ref	2.180(1.324,3.591)	3.437(2.222,5.316)	4.335(2.671,7.038)	< 0.0001	
49 ~ 63	ref	1.751(1.044,2.937)	1.863(1.213,2.861)	3.084(1.944,4.892)	< 0.0001	
64 ~ 85	ref	0.777(0.420,1.439)	1.113(0.651,1.904)	1.547(0.903,2.650)	0.008	
Race						0.092
Black	ref	2.096(1.431,3.069)	2.240(1.598,3.142)	2.246(1.438,3.509)	< 0.0001	
White	ref	1.796(1.263,2.553)	2.595(1.969,3.420)	3.975(2.892,5.463)	< 0.0001	
Other	ref	2.151(1.410,3.283)	3.195(2.158,4.731)	4.584(3.069,6.847)	< 0.0001	
Sex						0.167
Female	ref	2.195(1.316,3.663)	3.338(2.145,5.195)	3.787(2.384,6.016)	< 0.0001	
Male	ref	1.469(1.072,2.014)	2.023(1.515,2.702)	3.369(2.573,4.411)	< 0.0001	
BMI						0.853
Obese	ref	1.637(1.033,2.593)	1.948(1.347,2.817)	2.422(1.683,3.486)	< 0.0001	
Normal	ref	1.054(0.608,1.827)	1.085(0.611,1.927)	1.397(0.620,3.147)	0.486	
Overweight	ref	1.582(1.057,2.368)	1.691(1.117,2.560)	2.060(1.479,2.871)	< 0.001	

The data are presented as odds ratios (ORs), 95% confidence intervals (CIs), and *P* -values for trends

Adjusted for hypertension, CVD, smoking status, alcohol use, and DM

## Discussion

A previous study assessed relationship between the Tyg index and OSA by using the NHANES 2005–2008 dataset. However, prior research has focused mainly on investigating OSA as a standalone condition, thus neglecting the impact of the TyG index on the clinical presentations of OSA.

In this study, weighted linear regression and logistic regression analyses were employed to explore the correlation between the TyG index and OSA, encompassing OSA-related symptoms. Furthermore, a receiver operating characteristic curve was constructed to predict the risk of OSA. Following this design, a restricted cubic spline analysis was utilized to evaluate the relationship between the TyG index and OSA, along with its associated symptoms.

The primary finding of this study was that the TyG index exhibited a strong ability to predict OSA, with an AUC of 0.701 (95% CI: 0.6619–0.688). A higher AUC indicated that the model was more accurate at predicting the correct class, with 1 representing the optimal score. A model showing an AUC ranging between 0.7 and 0.8 signifies commendable performance. Additionally, the TyG index demonstrated a close association with OSA in both the crude model (OR = 0.24; 95% CI: 0.22–0.26) and Model 2 (OR = 0.02; 95% CI: 0.00–0.04). A higher TyG index indicated an increased risk of OSA (OR = 1.45; 95% CI: 1.02–2.42). Another significant finding was the correlation between the TyG index and symptoms in OSA patients experiencing breathing cessation, although its association with other symptoms remains inconclusive.

Insulin resistance is characterized by compromised glucose uptake, diminished glycogen synthesis, and reduced inhibition of lipid oxidation. Hence, when defining insulin resistance, it is crucial to consider not only triglycerides but also glucose levels. The TyG index is calculated based on fasting triglyceride and glucose levels. The incorporation of both lipid and sugar metabolism provides a straightforward approach for evaluating insulin resistance and the risk of metabolic syndrome. Moreover, several composite lipid indices are linked to OSA.

The lipid accumulation product (LAP), which was introduced in 2005, integrates waist circumference with triglyceride levels to provide insights into metabolic health and fat accumulation. Its robust ability to predict cardiovascular risks and diverse metabolic conditions is notable. However, the complexity of LAP measurements, which rely on accurate waist circumference data, poses challenges. The reliability of these measurements can be influenced by factors such as measurement technique, operator skill, and physiological variations such as postprandial distension. Although LAP emphasizes lipid metabolism, it may place less emphasis on sugar metabolism than the TyG index.

In 2010, the Visceral Adiposity Index (VAI) was acknowledged for its substantial correlations with cardiovascular diseases and metabolic syndrome [32]. The visceral fat index is computed by incorporating measurements such as waist circumference, weight, height, triglycerides, and systolic blood pressure. Its main objective is to evaluate the level of visceral fat accumulation (specifically, the degree of abdominal obesity). Research into the relationship between the VAI and OSA, including studies conducted by Mazzuca and a comprehensive Chinese study, has not identified a significant connection [33, 34]. The calculation of the VAI is more difficult than that of the TyG index. Therefore, the VAI is considered to be inferior to the TyG index.

The atherogenic index of plasma (AIP), which is a marker that reflects the esterification rate of HDL particles, provides a comprehensive understanding of the relationship between HDL-C and triglyceride levels [35]. The AIP is calculated by using easily accessible parameters, and studies have shown that the AIP is elevated in patients with OSA and is linked to disease severity [36]. Nevertheless, research suggests that the AIP increases in OSA only in individuals with moderate to severe disease and in those who have concurrent hypertension and diabetes [37]. In specific populations, such as individuals with exceptionally high or low triglyceride levels, the accuracy of the AIP may be compromised. Consequently, the clinical applicability of this marker could be limited [38].

The TyG index primarily evaluates insulin resistance and the risk of metabolic syndrome. The VAI predominantly assesses visceral fat accumulation and abdominal obesity. Moreover, the AIP is mainly associated with the risk of atherosclerosis. The LAP is primarily correlated with insulin resistance, metabolic syndrome, and the risk of cardiovascular disease.

Hence, although other indices demonstrate strong predictive abilities for OSA, the TyG index retains unique advantages. A recent meta-analysis aligns with this viewpoint, thus suggesting that the diagnostic accuracy of the TyG index is similar to that of other anthropometric indices [23].

The results of this study are consistent with those of a study conducted in Korea showing that an elevated TyG index is associated with an increased risk of developing OSA [39]. Nevertheless, a separate cross-sectional study suggested that the TyG index may not have a significant association with OSA [24]. This discrepancy could be attributed to the sample size; specifically, a larger sample size enhances the statistical power of a study, thus increasing its ability to accurately detect effects. In contrast, a small sample size may result in the study failing to detect significant relationships or differences, thus potentially leading to false-negative outcomes. In such instances, the study could underestimate the true associations between variables. A larger sample size allows for more precise estimations of population parameters, such as the mean, ratio, and effect size. Conversely, a smaller sample size may introduce larger sampling errors, thus causing sample statistics to deviate significantly from population parameters and reducing the credibility of the study results [40]. In contrast, this discrepancy could be linked to the definition of OSA. The NHANES database comprises a wide range of questionnaires, thus leading different researchers to choose varying questionnaires based on diverse standards, which could potentially introduce biases into the results. The diagnostic criteria for OSA that were employed in this study were carefully formulated by considering a range of clinical symptoms and other factors, thus ensuring a high level of credibility [25, 26]. Research by Andras Bikov et al. [41] indicated that the TyG index independently influences OSA. In This study, even after accounting for covariates, the TyG index maintained a strong correlation with OSA, thus confirming its independent effect. Additionally, findings from Andras Bikov’s study underscore a significant link between BMI and the TyG index, which is particularly prominent in individuals with higher BMIs. This study conducted a BMI-stratified analysis, and the observed results were consistent with the abovementioned research, thus emphasizing a stronger association between the TyG index and OSA among individuals in the higher BMI range. Among the clinical symptoms of OSA, the TyG index exhibited a stronger correlation with breathing cessation than with snoring and daytime sleepiness. Breathing cessation, which is a key clinical manifestation of OSA, has a profound impact on patients and is characterized by episodes of intermittent hypoxia [42]. Lin et al. [43] reported that in nonobese individuals, nocturnal hypoxia was notably linked to elevated triglyceride levels compared to those in the control group, which is consistent with the results of the current study. Additionally, in animal studies conducted by Li and colleagues, a marked increase in the expression levels of crucial transcription factors was observed in lean mice exposed to intermittent hypoxia. These transcription factors play essential roles in triglyceride biosynthesis, thus suggesting a potential mechanism through which intermittent hypoxia disrupts lipid metabolism [44]. Several animal studies have highlighted a potential association between intermittent hypoxia and the development of insulin resistance in lean mice. Insulin plays a crucial role in inhibiting triglyceride synthesis in the liver. The severity of intermittent hypoxia correlates with the degree of insulin resistance, thus resulting in a higher TyG index. Another study suggested that intermittent hypoxia can trigger insulin resistance and glucose intolerance, thus aggravating lipid accumulation in liver tissues [45]. In a small clinical trial involving human subjects, individuals with OSA exhibited dysregulated triglyceride metabolism, which improved with continuous positive airway pressure treatment [46]. Another study also indicated that intermittent hypoxia could impact the TyG index. In addition to affecting circulating cholesterol levels, OSA may regulate lipid metabolism by stimulating the generation of oxidatively stressed dysfunctional lipids, thus consequently influencing the TyG index [47].

This study has significant implications for clinical practice, given the increasing annual incidence of cardiovascular and cerebrovascular diseases attributed to OSA [48]. OSA has emerged as a significant health concern affecting human well-being. However, diagnosing OSA is often time-consuming, labor-intensive, and financially burdensome for patients. Hence, there is an urgent clinical need to identify a convenient and efficient diagnostic method for OSA. The TyG index, which is a cost-effective and easily measurable indicator, effectively meets these clinical requirements. This study’s findings offer valuable guidance to health care providers in promptly and conveniently assessing patients' risks of developing OSA. Given the limited data on the relationship between the TyG index and OSA risk in the field of sleep medicine, this research could offer essential insights that are applicable to high-risk adult populations for OSA.

Subgroup analysis plays a crucial role in scientific research, thus offering valuable insights into specific population subsets and enhancing the understanding of complex relationships within the data [49]. In this study, age, sex, and race were employed as stratification variables for subgroup analysis. The results suggested that younger Caucasian females may be at a greater risk for obstructive sleep apnea. (OSA) Age was identified as being a significant factor influencing OSA incidence, particularly within the 18–34 age group, where the triglyceride-glucose (TyG) index exhibited the most pronounced impact on OSA incidence.

### Strengths and limitations of the study

This study possessed several strengths that enhance its validity and reliability. First, the utilization of the NHANES database ensured sample diversity and representativeness, with strict adherence to the database guidelines. Second, the study employed linear, nonlinear, and logistic regression models to explore the relationship between OSA and the TyG index. Third, rigorous statistical adjustment techniques were applied to effectively control for any residual confounding factors potentially influencing the TyG index. Fourth, the use of the ROC curve illustrated that the TyG score serves as a robust predictive indicator for OSA. Fifth, the incorporation of subgroup analysis and interaction tests significantly bolstered the validity and reliability of the research findings. Notably, the subgroup analysis demonstrated that a younger average age was linked to a more substantial impact of the TyG index on OSA incidence.

Although this study had numerous strengths, the identification of certain limitations is crucial. First, the analytical and cross-sectional design of this study may weaken the evidence for the association between exposure and outcome. Future follow-up investigations are warranted to validate these findings. Additionally, diagnosing OSA by utilizing features from the NHANES database may introduce some bias. Furthermore, incomplete consideration of certain confounding factors, such as medication use, raises concerns. Finally, the generalizability of these findings to other regions is uncertain, thus highlighting the necessity for additional research.

## Conclusions

This study demonstrated a strong association between an elevated TG index and OSA. The TyG index is a novel indicator of insulin resistance that deviates from traditional assessment criteria. Due to its simplicity, cost-effectiveness, and reliability, it holds potential for broad application in primary health care settings and communities. The TyG index can serve as an independent predictor of OSA, which can help to detect and diagnose OSA early, thus reducing the threats posed by OSA. The findings emphasize the significance of integrating TyG assessments into clinical evaluations to inform targeted interventions and enhance outcomes for individuals at risk of OSA.

## Availability data and materials

The data that support the findings of this study are openly available in [NHANES] at (https://www.cdc.gov/nchs/nhanes/index.htm).

## Abbreviations

OR: Odds ratio
TyG: Triglyceride glucose
OSA: Obstructive sleep apnea
CVD: Cardiovascular disease
DM: Diabetes mellitus
NHANES: National Health and Nutrition Examination Survey
BMI: Body mass index
MAPI: Multivariate apnea prediction index
ROC: Receiver operating characteristic
AUC: Area under the curve
PIR: Poverty income ratio
FBG: Fasting blood glucose
TG: Triglyceride
VAI: Visceral adiposity index
AIP: Atherogenic index of plasma
LAP: Lipid accumulation product
